# An Immunomodulatory Gallotanin-Rich Fraction From *Caesalpinia spinosa* Enhances the Therapeutic Effect of Anti-PD-L1 in Melanoma

**DOI:** 10.3389/fimmu.2020.584959

**Published:** 2020-11-18

**Authors:** Paola Lasso, Alejandra Gomez-Cadena, Claudia Urueña, Alena Donda, Amaia Martinez-Usatorre, Pedro Romero, Alfonso Barreto, Susana Fiorentino

**Affiliations:** ^1^ Grupo de Inmunobiología y Biología Celular, Pontificia Universidad Javeriana, Bogotá, Colombia; ^2^ University of Geneva, Department of Pathology and Immunology, Geneva, Switzerland; ^3^ University of Lausanne, Department of Fundamental Oncology, Lausanne, Switzerland; ^4^ Swiss Federal Institute of Technology Swiss Institute for Experimental Cancer Research, Lausanne, Switzerland

**Keywords:** breast cancer, combined therapy, immunotherapy, natural products, melanoma, P2Et extract, PD-L1

## Abstract

PD-1/PD-L1 pathway plays a role in inhibiting immune response. Therapeutic antibodies aimed at blocking the PD-1/PD-L1 interaction have entered clinical development and have been approved for a variety of cancers. However, the clinical benefits are reduced to a group of patients. The research in combined therapies, which allow for a greater response, is strongly encouraging. We previously characterized a polyphenol-rich extract from *Caesalpinia spinosa* (P2Et) with antitumor activity in both melanoma and breast carcinoma, as well as immunomodulatory activity. We hypothesize that the combined treatment with P2Et and anti-PD-L1 can improve the antitumor response through an additive antitumor effect. We investigated the antitumor and immunomodulatory activity of P2Et and anti-PD-L1 combined therapy in B16-F10 melanoma and 4T1 breast carcinoma. We analyzed tumor growth, hematologic parameters, T cell counts, cytokine expression, and T cell cytotoxicity. In the melanoma model, combined P2Et and anti-PD-L1 therapy has the following effects: decrease in tumor size; increase in the number of activated CD4^+^ and CD8^+^ T cells; decrease in the number of suppressor myeloid cells; increase in PD-L1 expression; decrease in the frequency of CD8^+^ T cell expressing PD-1; improvement in the cytotoxic activity of T cells; and increase in the IFN**γ** secretion. In the breast cancer model, P2Et and PD-L1 alone or in combination show antitumor effect with no clear additive effect. This study shows that combined therapy of P2Et and anti-PD-L1 can improve antitumor response in a melanoma model by activating the immune response and neutralizing immunosuppressive mechanisms.

## Introduction

Cancer is a major public health problem and remains a main cause of mortality and morbidity worldwide. Cancer patients have traditionally been treated with chemotherapy and radiotherapy despite their significant toxicity and lack of effectiveness in all patients. In the last decade the role of the immune system in the control of tumor growth and progression has been well established, and several immunotherapies have been designed. Cancer vaccines, antibody-mediated immune modulation, adoptive T-cell transfer (1), and strategies to induce immunogenic cell death (2) have been tested in patients. However, to date immunotherapy has shown durable clinical benefit in only a small subset of patients. Consequently, the search for alternative treatments, therapies or combined strategies against cancer is highly important (3).

Antibodies against immune-checkpoint molecules, as programmed cell death protein-1 (PD-1), aim to neutralize immunosuppression of tumor infiltrating lymphocytes (TILs) have been approved for a variety of cancers (4–7). Unfortunately, only a minority of patients benefit from this checkpoint blockade across many types of cancer. These checkpoint inhibitors seem to be more effective when there has been a prior immune system activation associated with a sufficient number of TILs and a higher expression of PD-1 ligand (PD-L1) on tumor cells (4, 5, 8, 9). Thus, to improve the number of patients who benefit from PD-1 blockade, PD-1/PD-L1 antibodies are being used together and/or in combination with other anticancer agents or immunotherapies. The U.S. Food and Drug Administration (FDA) has approved the uses of atezolizumab (a monoclonal anti-PD-L1 antibody) in combination with albumin-linked paclitaxel (nab-Paclitaxel) for the treatment of patients with unresectable locally advanced or metastatic triple-negative breast cancer tumors expressing PD-L1 (10).

In our previous studies, we got a gallotannin-rich extract from *Caesalpinia spinosa* (P2Et) that has been previously reported to have antitumor activity in murine melanoma and breast cancer models (11–13). *Caesalpinia spinose*, commonly called Dividivi or Tara has been traditionally used by Colombian indigenous located on the Caribbean coast. It is a shrub with a pantropical distribution in forests, savannas, and semi-deserts. Dividivi fruits have 40 to 60% of hydrolyzable tannins with gallic acid as the main constituent. Tannic acids present in Dividivi inhibit the growth of tumors induced by chemical agents (14) and the carcinogenesis induced by UV light in mice (15). Also, gallic acid shows antioxidant, anti-allergenic, anti-mutagenic, anti-carcinogenic, and anti-inflammatory properties (16) and decreased proliferation of cervical cancer cells, leukemia, and melanoma (17, 18).

In our group, an extract derived from *Caesalpinia spinosa* called P2Et was obtained, which was chemically normalized and is now produced under good manufacturing practices (GMP) in LABFARVE Labs. Multiple esters derived from gallic acid have been reported, such as ethyl gallate, methyl gallate, ethyl 4,5-digaloyl quinate, methyl 4,5-digaloyl quinate, ethyl 3,5-digaloyl quinate, 4,5-digaloylquinic, ethyl 3,4,5-trigaloyl quinate, ethyl 5-galloyl quinate, among others in this extract (19).

In our hands, P2Et extract induces immunogenic cell death, displaying calreticulin on the cell surface, and ATP secretion in both breast and melanoma models (19). Additionally, in B16-F10 melanoma model we demonstrated that P2Et’s antitumor activity is partially abolished in immunodeficient mice, indicating that the antitumor activity of the P2Et treatment is highly dependent on the immune system (11). The treatment of C57BL/6 or BALB/c healthy mice with P2Et increased the number of CD4^+^ and CD8^+^ activated T, NK, regulatory T, dendritic and MDSC cells in lymphoid organs. However, in tumor-bearing animals, P2Et decreased the number of intratumor myeloid-derived suppressor cells (MDSCs) and increased the number of CD4^+^ and CD8^+^ T cells (20), suggesting a role in the modulation of the immune response, which is different in relation to the presence or not of tumors. According to the above studies, we hypothesize that the combined treatment with P2Et extract and anti-PD-L1 can improve the antitumor response through an additive antitumor effect.

In the present study, we investigated the antitumor and immunomodulatory activity of P2Et and anti-PD-L1 combined therapy in two different murine models, B16-F10 melanoma and 4T1 breast carcinoma. To this aim, we analyzed tumor growth, hematologic parameters, T cell counts, cytokine expression, and T cell cytotoxicity. Overall, we found that combined therapy with P2Et and anti-PD-L1 improves the antitumor response in the melanoma model by activating the immune response and neutralizing immunosuppressive mechanisms. In contrast, and surprisingly, no additive effect of the combination was observed in the breast cancer model.

## Materials and Methods

### Plant Material

*Caesalpinia spinosa* pods were collected in Villa de Leyva, Boyacá, Colombia and identified by Luis Carlos Jiménez from the Colombian National Herbarium (voucher specimen number COL 523714, Colombian Environmental Ministry agreement number 1470 related to the use of genetic resources and derived products). The P2Et extract was produced under GMP conditions and chemically characterized as previously described (19, 21).

### Mice

Young (6 to 12 weeks old) female C57BL/6 and BALB/c mice were purchased from the Jackson Laboratories (Bar Harbor, ME, USA) and housed at the animal facilities of the Pontificia Universidad Javeriana (PUJ, Bogotá, Colombia) following the established protocols of the Ethics Committee of the Faculty of Sciences, PUJ, and National and International Legislation for Live Animal Experimentation (Colombia Republic, Resolution 08430, 1993; National Academy of Sciences, 2010). The present study was approved by the ethics committee of the Faculty of Sciences, PUJ, on August 9, 2018. Each specific protocol was also approved by the animal experimentation committee of PUJ. Mice were maintained in polyethylene cages with food and water provided *ad libitum*, on a 12-h light/dark cycle at 20–22°C and 40–60% humidity.

### Tumor Cell Lines and Culture Conditions

B16-F10, 4T1, Mel-Rel, TS/A, MCF-7, MDA-MB-468, LAU 145, and Me290 cell lines were cultured in RPMI-1640 (Eurobio, Toulouse, France) supplemented with 10% heat-inactivated fetal bovine serum (FBS), 2 mM L-glutamine, 100 U/ml penicillin, 100 μg/ml streptomycin, 0.01 M HEPES buffer, and 1 mM sodium pyruvate (Eurobio) and incubated in a humidified environment at 37°C and 5% CO_2_. Cells were grown until 75% confluency and passaged using trypsin/1× EDTA (Eurobio), washed with PBS, and resuspended in supplemented RPMI-1640.

### Abs

The following Abs were used for cell surface staining: anti-CD3 Pacific Blue (clone 17A2), anti-CD8 PE Dazzle (clone 53.6.7), anti-CD45 PE-Cy5 (clone 30-F11), anti-Ly-6G PE-Cy7 (clone 1A8), anti-Ly-6C APC-Cy7 (clone AL-21), anti-PD-L1 PE (clone 10F.9G2), anti-PD-1 APC (clone 29F-1A12), CD11b Alexa Fluor 700 (clone M1/70), B220 PE (clone RA-3-6B2) (Biolegend, San Diego, CA, USA), anti-CD45 PE (clone 30-F11), anti-CD4 PerCP (clone RM4-5), Gr1 PE-Cy7 (clone RB6-8C5), CD11c FITC (clone HL3), and anti-CD44 APC (clone IM7) (BD Biosciences, San José, CA, USA). A LIVE/DEAD Fixable Aqua Dead Cell Stain Kit (Life Technologies, Thermo Scientific, Eugene, OR, USA) was used for dead cell exclusion.

### *In Vitro* Induction of PD-L1

B16-F10 and 4T1 cells (1 × 10^5^) were treated with half of P2Et IC_50_ (50% inhibition of cell growth), 100 mM of cobaltous chloride (CoCl_2_, positive control) or ethanol (EtOH, negative control) for 6, 12, 24, or 48 h. Then, cells were harvested with TRIzol reagent (Life Technologies Corporation, Invitrogen, NY, USA) and stored at −80°C until further processing. In addition, cells were labeled with anti-PD-L1 PE and analyzed by flow cytometry.

### Reverse Transcription–Polymerase Chain Reaction

Total RNA from B16-F10 or 4T1 treated cells *in vitro*, tumor cells from melanoma or 4T1 tumor-bearing mice or skin from healthy C57BL6 or BALB/c mice, as control, was extracted using TRIzol LS reagent (Life Technologies Corporation) in accordance with the manufacturer’s instructions. Complementary DNA (cDNA) was synthetized from total RNA with SuperScripts III Reverse Transcriptase, following manufacturer’s instructions (Life Technologies Corporation, Invitrogen, NY, USA). For real-time PCR reaction, 600 ng of cDNA, DNA Master Plus SYBR Green I (Roche Applied Science, IN, USA) and 250 nM forward and reverse primers were added in a total volume of 20 µl. The following primers were used: murine PD-L1 (forward: CCATCCTGTTGTTCCTCATTG; reverse: CACTGCTTACGTCTCCTCG), human PD-L1 (forward: GTACCGCTGCATGATCAGCTAT; reverse: GGCATTGACTTTCACAGTAATTCG) (22); murine glyceraldehyde-3-phosphate dehydrogenase (GAPDH) (forward: TCAACAGCAACTCCCACTCTTCCA; reverse: ACCCTGTTGCTGTAGCCGTATTCA); and human *β*2-microglobuline (forward: TGGAGGCTATCCAGCGTACT; reverse: CGGCAGGCATACTCATCTTT). Reactions were performed in duplicates using QuantStudio 3 Real-Time PCR systems (ThermoFisher Scientific, Waltham, MA, USA). The relative expression level was normalized to murine GAPDH expression or human *β*2-microglobuline and calculated using the comparative CT method (2^−ΔΔCT^).

### *In Vivo* Tumor Development Experiments and Treatment

For melanoma tumor induction, C57BL/6 mice were subcutaneously (s.c.) inoculated in the right flank with 1 × 10^5^ viable B16-F10 cells. For the breast cancer murine model, 1 × 10^4^ viable 4T1 cells were s.c. injected into the right mammary fat pad of BALB/c mice. To evaluate the effect of treatments on tumor growth, 3–5 days after tumor cell inoculation, 5–7 mice per group were treated with 200 µg of anti-PD-L1 (clone: 10F.9G2; BioXcell, West Lebanon, NH, USA), 75 mg/kg (B16-F10 model), or 18.7 mg/kg (4T1 model) body weight of P2Et extract, anti-PD-L1 plus P2Et or PBS (negative control) two times per week. To ensure low toxicity, P2Et therapeutic dose was determined as a fourfold lower than the median lethal dose (LD-50) estimation (11). In all experimental settings, the size of the tumors was assessed three times per week with Vernier calipers, and the volume was calculated according to the formula V (mm^3^) = L (major axis) × W^2^ (minor axis)/2 (23). Mice were euthanized by CO_2_ inhalation, and then spleen, tumor-draining lymph nodes (TDLN), and tumor were removed and processed. Looking for sufficient statistical power adjusted to the standard deviation and to the proportion of losses in each model, six mice were included for each treatment group.

### Hematology

Approximately 700 µl of blood was collected by cardiac puncture immediately after euthanasia into tubes containing EDTA. A part of the blood was used to evaluate hematological parameters on the MICROS-60 hematology analyzer (Horiba ABX-Diagnostics, Montpellier, France). The remaining blood was used to separate plasma and evaluate cytokine levels.

### Cytokine Assay

Cytokine evaluation was performed using a Cytometric Bead Array (CBA) mouse Th1, Th2, Th17 cytokine kit (BD Biosciences) according to the manufacturer’s instructions. Experiments were performed twice, and each experiment was performed in duplicate. Events were acquired using a FACSAria II flow cytometer (BD Immunocytometry Systems), and the results were subsequently analyzed using FCAP array software version 3.0 (BD Biosciences). Data were log-transformed and plotted as the mean ± SEM.

### Cytometry

Briefly, 1 × 10^6^ cells were stained with LIVE/DEAD Fixable Aqua for 20 min in dark conditions at room temperature. After washing with PBS 2% FBS, the cells were stained for 30 min at 4°C in dark conditions with the surface antibodies at final concentration of 1 µg/ml according to the designed multicolor panels. Then, the cells were acquired by flow cytometry using a FACSAria II flow cytometer (BD Immunocytometry Systems, San José, CA, USA), and the results were subsequently analyzed using FlowJo 9.3.2 software (Tree star, Ashland, OR).

### Cytotoxicity Assay by Flow Cytometry, CFSE/7-Amino Actinomycin D

To expand tumor-specific cytotoxic T cells, splenocytes (3 × 10^6^) from each group of B16-F10 or 4T1 tumor-bearing mice were plated in 24-well plates. Cells were cultured in a total volume of 3 ml of RPMI-1640 (Eurobio) supplemented with 10% FBS (Eurobio), 2 mM L-glutamine, 100 U/ml penicillin, 100 μg/ml streptomycin, 0.01 M HEPES buffer, 1 mM sodium pyruvate (Eurobio), IL-2 (10 UI/ml), IL-7 (1 ng/ml), and B16-F10 or 4T1 cell lysate (20 µg/ml). The cells were then incubated in a humidified environment at 37°C and 5% CO_2_ for four days. Later, the cells were restimulated with the respective cell lysate (20 µg/ml) and cultured for three additional days. Then, cells were collected and resuspended in medium for the cytotoxicity assay. B16-F10 or 4T1 cells were labeled with carboxyfluorescein succinimidyl ester (CFSE; Thermo Fisher Scientific, MA, USA) at a final concentration of 1 µM for 20 min at 37°C following the manufacturer’s recommendations. After quenching, the labeling reaction was stopped by the addition of complete culture medium, followed by a 5-min incubation at 37°C. After 2 washes, the CFSE-labeled target cells were resuspended and used for the cytotoxicity assay. The cell concentration was adjusted to 5 × 10^5^ cells/ml, and 100 µl/well, and plated into 96-well plates. Splenocytes were added at 10:1 and 20:1 effector-target (E:T) ratios. The plates were incubated in a humidified atmosphere of 5% CO_2_ and 37°C. After 12 h, the wells were harvested and labeled with 7-amino actinomycin D (7-AAD) to stain dead cells. All cells in each tube were acquired on a FACSAria II instrument (BD Immunocytometry Systems) flow cytometer, and the results were analyzed using FlowJo software (Tree Star). Analysis was performed by gating on the target cells and measuring the 7-AAD-positive cells (24). Cells positive for both 7-AAD and CFSE were considered lysed. Additionally, we calculated the percentage of cell loss in each well assuming that the number of target cells read from the 0:1 effector–target ratio was 100% of events (25). This percentage was added to the percentage of dead cells, and the percentage of cytotoxic activity was calculated using the following equation:

$$Cytotoxicity\;(\%)=\frac{100x[deadtargetcells(\%)-spontaneousdeath(\%)]}{\left[100-spontaneous\;death\;(\%)\right]}$$

### Statistical Analysis

Statistical analysis of the significance between two groups was calculated using the Mann–Whitney *U* test. Differences among subject groups were evaluated using Kruskal–Wallis and Dunn’s posttest for multiple comparisons. For all cases, the differences were considered statistically significant when *p* < 0.05. GraphPad Prism version 6.0 for Mac OS X statistics software (GraphPad Software, San Diego, CA) was used for the statistical analyses.

## Results

### PD-L1 Expression Is Modulated by P2Et Extract in B16-F10 and 4T1 Cells

**Figure 1:**
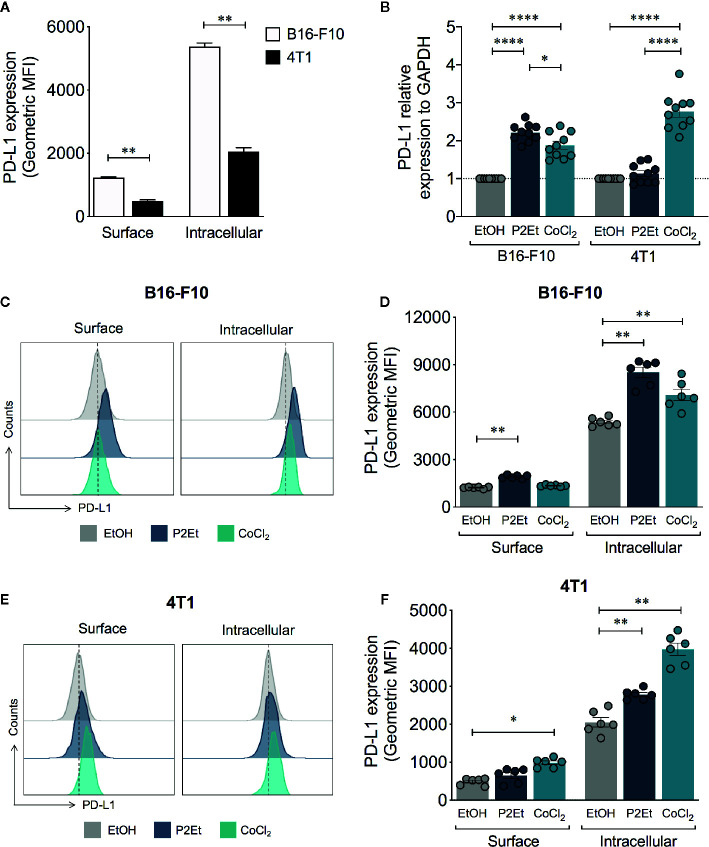
P2Et treatment modulates the expression of PD-L1 on B16-F10 and 4T1 cells. **(A)** Geometric Mean Fluorescence Intensity (MFI) of surface or intracellular PD-L1 expression in B16-F10 and 4T1 cells after 48 h. **(B)** PD-L1 relative expression to GAPDH gene in B16-F10 and 4T1 cultured cells during 48 h with one-half of the P2Et IC_50_ extract, cobaltous chloride (CoCl_2_) or ethanol (EtOH) analyzed by qRT-PCR and quantitated as a fold change against the control by using the 2^−ΔΔCT^ method. Representative histograms of surface and intracellular PD-L1 expression in **(C)** B16-F10 and **(E)** 4T1 cells treated during 48 h with one-half of the P2Et IC_50_, CoCl_2_ or ethanol. Geometric MFI of surface and intracellular PD-L1 expression in **(D)** B16-F10 and **(F)** 4T1 cells treated. In all cases data are represented as the mean ± SEM of at least three independent experiments. The *p* values were calculated using a Mann–Whitney *U* test or Kruskal–Wallis test with Dunn’s post-test. *p < 0.05, **p < 0.01, ****p < 0.0001.

In order to assess the impact of P2Et treatment on tumor PD-L1 expression, B16-F10 and 4T1 tumor cells were treated with sub-lethal concentration of the extract (1/2 IC_50_), for variable exposure times. We observed an upregulation of protein over time at transcriptional or protein level, being the most significant at 48 h of treatment (**Supplementary Figure 1**). In basal conditions, B16-F10 cells exhibited higher levels of surface and total (surface and intracellular) PD-L1 expression compared to 4T1 cells (**Figure 1A**). The analysis of the gene expression of PD-L1 showed that P2Et induces the transcription of PD-L1 mRNA in B16-F10 but not in 4T1 (**Figure 1B**). Analysis done after 48 h of stimulation, confirmed that surface and intracellular PD-L1 expression on B16-F10 cells are higher after P2Et treatment, compared with 4T1 (**Figures 1C-F**). When comparing P2Et with the positive control CoCl_2_, a hypoxia inducer that increases PD-L1 (26), it was observed that at least in B16-F10, P2Et increases PD-L1 by a different signaling pathway possibly independent of HIF-1*α*, (**Figures 1B–D**). To determine whether the P2Et-mediated modulation of PD-L1 could be generalized or is specific to these cell lines, we assessed PD-L1 expression on the murine melanoma cell line (Mel-Rel), the murine breast cancer cell line (TS/A), the human melanoma (LAU 145 and Me 290), and the human breast (MCF-7 and MDA-MB-468) cancer cell lines. The increase in PD-L1-mRNA levels induced by P2Et was observed in all human cell lines but not in TS/A and minimally in Mel Rel murine cell lines (**Supplementary Figure 1G**). In contrast, the surface level of PD-L1 was increased on TS/A, MCF-7 and Me 290 cell lines but, decreased in MDA-MB-468 and LAU 145, while the intracellular PD-L1 level was increased in all human cell lines but not in murine cell lines (**Supplementary Figure 1H**). These results suggest that the effects of P2Et differ depending on the cell line, regardless of tumor type and origin.

### P2Et Treatment Enhances the Response to *α*PD-L1 immunotherapy in B16-F10 Tumor-Bearing Mice

**Figure 2:**
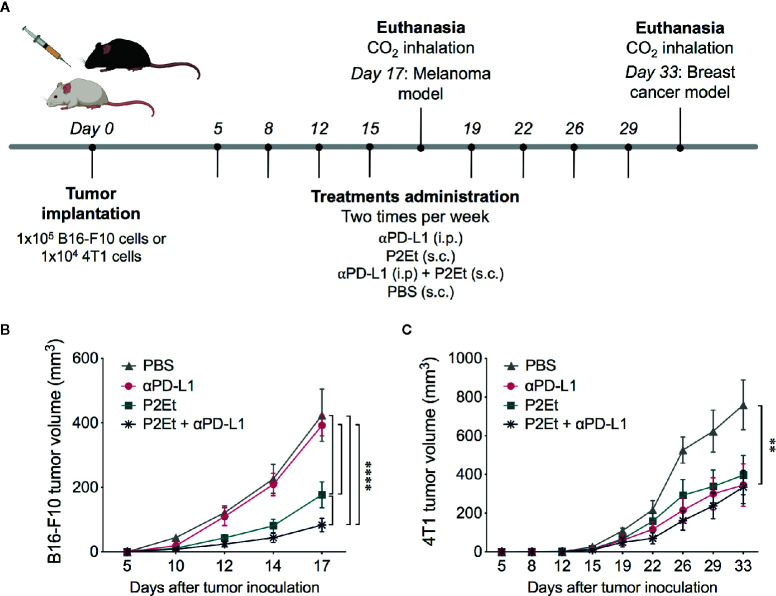
P2Et treatment enhances the response to anti-PD-L1 immunotherapy in B16-F10 tumor-bearing mice. **(A)** Experimental treatment scheme. Tumor was established by injection of B16-F10 or 4T1 cells and 5 days after tumor cell injection, treatments were administrated two times per week for 17 and 33 days, respectively. **(A)** Tumor volume in B16-F10 tumor-bearing mice treated with *⍺*PD-L1, P2Et, P2Et plus *α*PD-L1 or PBS. **(B)** Tumor volume in 4T1 tumor-bearing mice treated with each treatment. In all cases data are represented as the mean ± SEM (n = 6). The *p* values were calculated using a two-way ANOVA test. **p < 0.01, ****p < 0.0001.

In addition to the properties of P2Et extract to induce immunogenic cell death and to modulate the immune response (11–13, 20), it also upregulates the PD-L1 expression in certain cell lines, which could sensitize cancer cells to the anti-PD-L1 antibody. To check this hypothesis, we tested a combined therapy of P2Et and anti-PD-L1 (*α*PD-L1) in B16-F10 tumor-bearing mice (**Figure 2A**). 1 × 10^5^ B16-F10 cells were injected s.c to C57BL/6 mice, and 5 days after, mice were treated with P2Et, *α*PD-L1, P2Et plus *α*PD-L1 or PBS. Mice treated with P2Et plus *α*PD-L1 showed the smallest tumor size compared to the other groups, even when compared to the P2Et only treated mice, while therapy with only *α*PD-L1 did not impair tumor growth compared to the PBS control group (**Figure 2B**). In addition, mice treated with P2Et plus *α*PD-L1 showed a higher percentage or survival (**Supplementary Figure 3**).

In order to evaluate whether the modulation of PD-L1 on the tumor cell could be related to a possible adjuvant activity of PD-L1 *in vivo*, we evaluated the effect of combined therapy of P2Et and *α*PD-L1 in 4T1 tumor-bearing mice (**Figure 2A**). Mice treatment with P2Et delayed tumor growth as previously observed (12, 20), but although *α*PD-L1 and P2Et plus *α*PD-L1 treatments, as P2Et alone, had a positive effect on tumor growth, no synergistic activity was observed on 4T1 tumor-bearing mice (**Figure 2C**).

### P2Et Treatment Alone or in Combination with *α*PD-L1 Maintains Hematological Parameters in Normal Ranges in B16-F10 Melanoma Model

**Figure 3:**
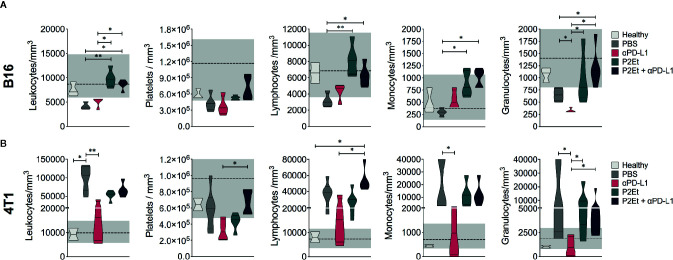
Hematological parameters. Hematological parameters of B16-F10 **(A)** or 4T1 tumor-bearing mice **(B)**. Data are represented as violin plots showing the frequency distribution of the data, median, and quartiles. The gray area shows the normal reference range for each mouse strain according to the breeding company, and the dotted line corresponds to the median of the reference range. *p < 0.05, **p < 0.01.

P2Et extract has an important antioxidant activity (19) that could protect host cells from oxidative stress during cancer expansion and progression. Thus, we evaluated hematological parameters in normal and treated tumor-bearing mice. In the B16-F10 model, there was no change in leukocytes, platelets, lymphocytes, monocytes, and granulocytes’ counts in mice treated with P2Et alone or in combination with *α*PD-L1 compared with healthy mice (**Figure 3A**). Mice treated with P2Et or P2Et plus *α*PD-L1 had a higher number of leukocytes, lymphocytes, monocytes, and granulocytes compared to the untreated mice. However, in the 4T1 model, the only populations that remained in the normal range were platelets in mice treated with P2Et plus *α*PD-L1, and monocytes and granulocytes in mice treated with *α*PD-L1 (**Figure 3B**). In conclusion, a significant recovery of hematological parameters was evidenced in melanoma model when P2Et alone or in combination with *α*PD-L1 was used.

### P2Et Treatment Increases PD-L1 Expression in Tumor Cells *In Vivo*

**Figure 4:**
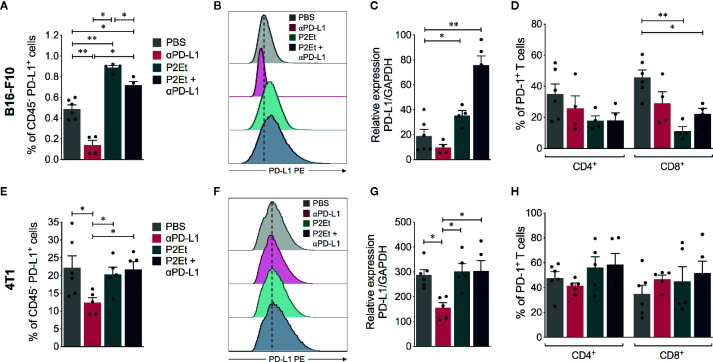
PD-L1 and PD-1 expression in melanoma and breast tumor infiltrating immune cells. **(A)** Frequency of CD45 negative cells expressing PD-L1 in mice treated with *⍺*PD-L1, P2Et, P2Et plus *⍺*PD-L1 or PBS (control). **(B)** MFI of PD-L1 expression in CD45 negative cells. **(C)** PD-L1 relative expression to GAPDH gene in tumor cells from each mice group analyzed by qRT-PCR and quantitated as a fold change against the control (healthy mice) by using the 2^−ΔΔCT^ method. **(D)** Frequency of intratumoral CD4^+^ and CD8^+^ T cells expressing PD-1. CD4^+^ and CD8^+^ T cells were selected from live CD3^+^ CD45^+^ cells. **(E)** Frequency of CD45 negative cells expressing PD-L1 in 4T1 tumor-bearing mice treated with *⍺*PD-L1, P2Et, P2Et plus *⍺*PD-L1 or PBS (control). **(F)** MFI of PD-L1 expression in CD45 negative cells from 4T1 tumor-bearing mice. **(G)** PD-L1 relative expression to GAPDH gene in tumor cells from each 4T1 tumor-bearing mice group analyzed by qRT-PCR and quantitated as a fold change against the control (healthy mice) by using the 2^−ΔΔCT^ method. **(H)** Frequency of intratumoral CD4^+^ and CD8^+^ T cells expressing PD-1 in 4T1 tumor-bearing mice. In all cases data are represented as the mean ± SEM. *p < 0.05, **p < 0.01.

Given that the expression of PD-L1 in tumor cells may affect the response to immune blockade therapy, we evaluated PD-L1 expression in tumor CD45 negative cells recuperated from mice. In the melanoma model, P2Et extract alone or in combination with *α*PD-L1 significantly increased surface PD-L1, (**Figures 4A, B**) and PD-L1 mRNA expression in tumors (**Figure 4C**). In contrast, it significantly decreased the frequency of CD8^+^ T cells expressing PD-1 receptor (**Figure 4D**). This suggests that P2Et can improve the effector response of CD8^+^ T cells. P2Et treatment did not modulate the PD-L1 expression in the breast 4T1 model *in vivo* (**Figures 4E–G**) as previously shown (**Figure 1**) and neither did it modulate the frequency of T cells expressing PD-1 (**Figure 4H**). In both models, a significant decrease in PD-L1 surface expression after *α*PD-L1 treatment compared with other groups (**Figures 4A, B**, **E, F**) was found. However, there has been a diminution due to a masking effect of therapeutic *α*PD-L1 antibody (**Supplementary Figure 2**). This would explain the discrepancy between protein level and mRNA expression in the P2Et plus *α*PD-L1 group of B16-F10 model (**Figures 4A, C**). Recent studies have shown that adjacent cells around the tumor (dendritic or macrophages), express higher levels of PD-L1 and play an important role in the response to immunotherapy (8, 27, 28). Therefore, we assessed the PD-L1 expression also in tumor infiltrating CD45^+^ cells in both models, but no significant differences were found among the different treatments (data not shown).

### P2Et Treatment Alone or in Combination With *α*PD-L1 Modulates the Immune Response

**Figure 5:**
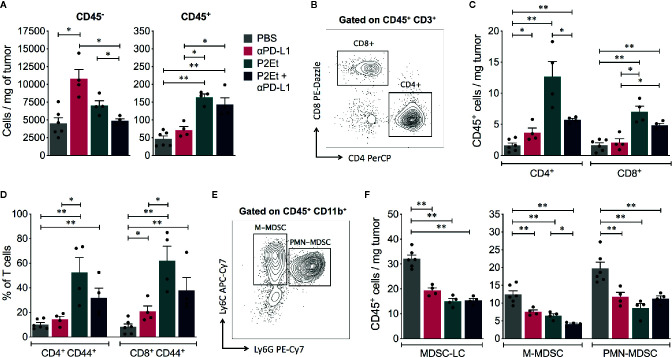
P2Et treatment alone or in combination with αPD-L1 modulates the immune response in melanoma B16-F10 model. **(A)** Absolute numbers of CD45^−^ and CD45^+^ cells per mg of tumor in mice treated with *⍺*PD-L1, P2Et, P2Et plus *⍺*PD-L1 or PBS (control). **(B)** Representative dot plots of the CD4^+^ and CD8^+^ T cell populations. **(C)** Absolute numbers of CD4^+^ and CD8^+^ T cells per mg of tumor in each group of treated mice. **(D)** Frequency of activated CD44^+^ CD4^+^ and CD8^+^ T cells in tumor from each group of treated mice. **(E)** Representative dot plots of monocytic MDSC (M-MDSC: CD11b^+^ Ly6C^hi^ Ly6G^−^) and polymorphonuclear MDSC (PMN-MDSC: CD11b^+^ Ly6C^lo^ Ly6G^+^). **(F)** Number of MDSC like cells (MDSC-LC), M-MDSC, and PMN-MDSC per mg of tumor from *⍺*PD-L1, P2Et, P2Et plus *⍺*PD-L1 or PBS treated mice. In all cases data are represented as the mean ± SEM. *p < 0.05, **p < 0.01.

The fine analysis of the type of cells present in the tumor allowed showing that the relationship between tumor cells and immune cells varies depending on the type of treatment. Therefore, in melanoma model the therapy with P2Et or P2Et plus *α*PD-L1 induces a significant increase in the number of tumor-infiltrating CD45^+^ cells per mg of tissue, compared to PBS or *α*PD-L1 groups (**Figure 5A**). Within the CD45^+^ cells, the population of CD4^+^ and CD8^+^ T cells was analyzed (**Figure 5B**) and an increase of cell numbers was found in mice treated with P2Et or with the combination (**Figure 5C**). In addition, P2Et or P2Et plus *α*PD-L1 treatment, also induced a high frequency of activated (CD44^+^) CD4^+^ and CD8^+^ T cells (**Figure 5D**). The assessment of other immune cell populations in the tumor (**Figure 5E**) showed that the three treatment strategies decreased the number of MDSC, which due to lack of functional evaluation are called myeloid-derived suppressor like cells (MDSC-LCs) (**Figure 5F**) (29). However, the number of tumor-infiltrating monocytic MDSC (M-MDSC) was lower with P2Et plus *α*PD-L1 treatment compared to P2Et alone (**Figure 5F**). Recently it was described that PD-L1 expression on dendritic cells (DCs) and macrophages correlated with the efficacy of immunotherapy in ovarian cancer and melanoma (30). We found that P2Et treatment increased the frequency of conventional DCs (cDCs) in TDLN and also increased PD-L1 expression in these cells and in macrophages (**Supplementary Figures 4A, B**), suggesting that the P2Et-based therapy alone may trigger counter regulatory immune loops, thus providing a rationale for combination with immune check point blockade. However, no differences were found in the spleen (**Supplementary Figure 5**).

Similar results were found in the 4T1 model. The treatment with P2Et, *α*PD-L1 or P2Et plus *α*PD-L1 significantly increased the number of CD45^+^ cells per mg of 4T1 tumor (**Supplementary Figure 6A**), with an increase in CD4^+^ and CD8^+^ T cells (**Supplementary Figure 6B**). Likewise, we found an increase in the frequency of activated CD44^+^ CD4^+^ and CD44^+^ CD8^+^ T cells when treated with P2Et plus *α*PD-L1, and an increase of CD44^+^ CD8^+^ T cells when treated with P2Et (**Supplementary Figure 6C**). Moreover, we found a significant decrease in the total number of MDSC-LC after all the treatments compared with non-treated mice, mainly due to the decrease in polymorphonuclear MDSC (PMN-MDSC) cells (**Supplementary Figure 6D**). Furthermore, we showed that P2Et plus *α*PD-L1 treatment increased the frequency of cDC, and P2Et alone increased their PD-L1 expression. However, in the macrophage population no differences were observed among groups (**Supplementary Figures 4C, D**).

### P2Et in Combination With *α*PD-L1 Enhances the Effector Response of Cytotoxic Cells in Melanoma and Breast 4T1 Model

**Figure 6:**
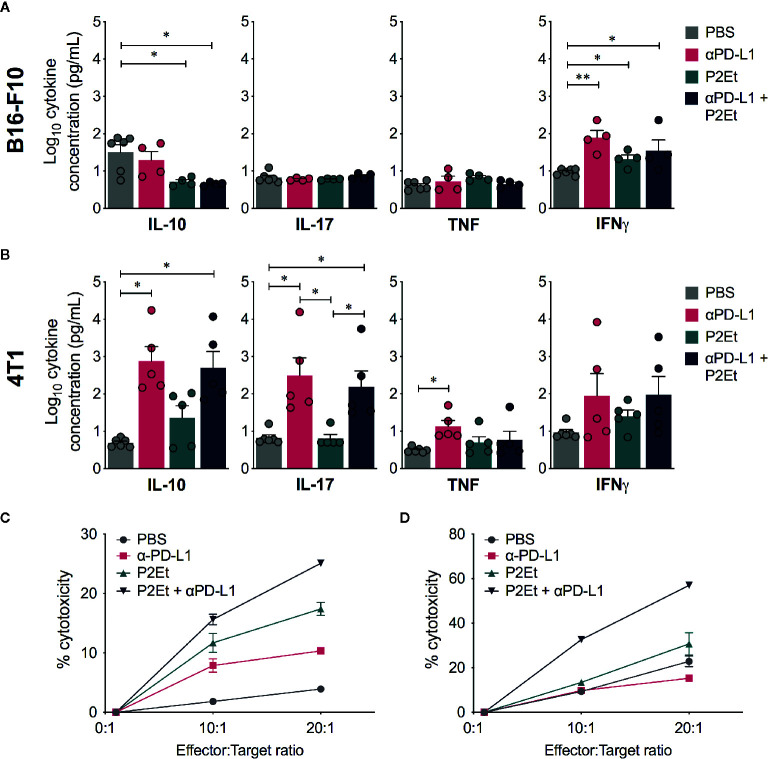
Effector response in melanoma and breast cancer. Cytokine levels in the serum of B16-F10 tumor-bearing mice **(A)** or 4T1 tumor-bearing mice **(B)** treated with *⍺*PD-L1, P2Et, P2Et plus *⍺*PD-L1 or PBS (control). The data were log-transformed and plotted as the mean ± SEM. **(C)** Cytotoxicity percentage of spleen cells from B16 tumor-bearing mice against B16-F10 melanoma cells at E:T ratios of 0:1, 10:1 and 20:1. **(D)** Cytotoxicity percentage of spleen cells from 4T1 tumor-bearing mice against 4T1 cells at E:T ratios of 0:1, 10:1 and 20:1. In all cases data are represented as the mean ± SEM. *p < 0.05, **p < 0.01.

To modulate the immune response in the B16-F10 melanoma model based on the known capacity of P2Et, we evaluated the production of cytokines in each group. Interestingly, a significant increase in the plasma concentrations of IFN**γ** was found in the *α*PD-L1, P2Et and P2Et plus *α*PD-L1 groups compared to the control group, while a significant decrease in the plasma concentrations of IL-10 was found when P2Et was used (**Figure 6A**). In contrast, a significant increase in the plasma concentrations of IL-10 and IL-17 was found in *α*PD-L1 or P2Et plus *α*PD-L1 treated 4T1-BALB/c mice as compared to B16-C57BL/c mice (**Figure 6B**) with a downward trend in IL-6 (data not shown), confirming our previous results (12). Taking into account the potential role of IL-10 and IL-17 in tumor promotion (31–33), these results may explain the differences in the treatment outcome in both models. No differences were found in the plasma concentrations of IL-4 and IL-2 (data not shown) between groups. Finally, we also assessed the cytotoxic capacity of spleen cells after expanding tumor-specific cytotoxic T cells, as explained in the *Materials and* *Methods* section. We found that P2Et plus *α*PD-L1 treatment induced a higher cytotoxic potential of cytotoxic cells both in melanoma and in the breast cancer model (**Figures 6C, D**), suggesting that combined therapy improves CD8^+^ T cell effector functions, which would entail a better response to immunotherapy.

## Discussion

In the current study, we evaluated the combined treatment of P2Et, a polyphenol-rich extract obtained from *Caesalpinia spinosa*, and *α*PD-L1 antibody for melanoma and breast cancer in mouse models. This in accordance with the fact that, in breast cancer, there is no high response to immunotherapy compared to that observed in other types of cancer such as melanoma, lung, kidney cancer, among others (34). We confirm the antitumor activity of P2Et both on melanoma B16-F10 and breast cancer 4T1 tumor models (11, 13). We then demonstrate that P2Et extract improves the response to treatment with *α*PD-L1 antibody in the murine melanoma model, finding that the combined therapy increases PD-L1 expression in tumor cells, maintains most hematological parameters in the normal range, modulates the immune response and enhances the effector response of cytotoxic cells. By contrast although experiments on the number of TILs and the effector response of cytotoxic cells revealed favorable results in the breast cancer model, combined therapy had no effects in tumor growth compared to individual therapy with P2Et or *α*PD-L1. These results reflect the necessity to evaluate individual heterogenicity of the response, and its relationship with the tumor type and genetic background of the individual.

We observed heterogenous responses of PD-L1 expression on different tumor cells treated with P2Et extract. The mechanisms governing the PD-L1 expression are not well understood (35). PD-L1 is not only expressed in tumors, but also on the surface of B lymphocytes, monocytes, natural killer cells, macrophages, and vascular endothelial cells (36). However, the mechanisms governing the expression on tumor cells and immune cells seem to be different. Different polyphenols have been evaluated in their ability to modulate the expression of PD-L1 or to synergize with other therapies (37, 38). This seems to depend on the microenvironment context, the dose of the compounds, and the type of cells to which they are directed (39). A recent study showed that two polyphenolic compounds, curcumin and apigenin, decrease the PD-L1 expression on melanoma cells *in vitro* and *in vivo* in a murine model, diminishing the tumor growth and increasing immune cell infiltration (40). Nonetheless, other studies have shown that an increase in PD-L1 expression may enhance therapeutic effect of *α*PD-L1 antibodies. In fact, treatments with resveratrol and piceatannol alone or in combination elicited an upregulation of PD-L1 in some breast and colorectal cancer cell lines (37).

In tumors, PD-L1 expression can be regulated genetically and epigenetically. Amplifications and deletions of the PD-L1 gene have been identified on primary tumor cells, and both have been related with worse prognosis of the disease (41). Complexity of anti-PD-L1 treatment effectivity is evidenced by the observation that in some melanoma patients with detection of PD-L1 by immunohistochemistry, a positive clinical response to anti-PD-L1 treatment has been observed, while a bad response have been also observed in spite of a strong PD-L1 expression (42, 43). PD-L1 levels may be upregulated by different intracellular signals as Akt activation or PTEN dysfunction (44) or, by regulation of micro-RNAs (45). Natural products act on these intracellular signals modulating several tumorigenic signals (46), having also effect on microRNAs. Therefore, the identification of mechanisms involved in PD-L1 modulation by P2Et or other natural products requires a holistic approach including integrate omics science and systems biology.

As we mentioned before, mechanisms implied in PD-L1 expression on tumor and immune cells seems to be different. The study of PD-L1 expression on non-small cell lung carcinoma (NSCLC), shows that reduced methylation of the PD-L1 promoter on tumor but not immune cells, increases the PD-L1 expression in response to external stimuli (47). Immune response by itself, regulates PD-L1 expression though interferon signaling (48) and even signals inducing epithelial-to-mesenchymal transition of tumor cells, improves tumor responses to anti-PD-L1 antibodies (49).

In this study, we show that P2Et modulates the gene expression of PD-L1, as well as their protein expression, and this differs among the different cell lines studied. Although the factors involved were not studied, it is important to highlight that while the B16-F10 cell line increased gene expression and the protein response to P2Et, the 4T1 line showed a significant increase only in the presence of CoCl_2_. However, our results suggest that while P2Et exerts an activating effect of PD-L1 expression on the B16-F10 cells, it has little to no influence in 4T1 cells. These differences appear to be related to the intrinsic differences of tumor cells, but also to the *in vivo* tumor response in different mice strains. Different mice strains may have different immune status and thus may display different immune infiltrates in tumors which in turn can influence the outcome of immunotherapy. Thus, Sellers and al., have been shown immune variations specific to the strain (50). The BALB/c mice are considered a Th2 prone strain, whereas a Th1 response characterizes the C57BL/6 mice. Nevertheless, this also depends on the type of stimulus (51). In this sense, the production of IL-17 could explain some differences between the strains. Although it is true that IL-17 has been correlated with tumor progression in the 4T1 and B16 models (52, 53), we did not observe important differences in the plasma levels of this cytokine in the B16 model, but in the 4T1 model. Interestingly, treatment with PD-L1 alone or accompanied by P2Et significantly increased IL-17 in 4T1 mice, but not in B16. The mechanisms that regulate the activation of LTh17 depend on complex orchestrations of cytokines in the tumor microenvironment and their function can be dual (54). For example, in breast cancer it has been clearly observed that IL-23 induced by PGE2 (55) plays a very important role in the recruitment and activation of LTh17 related to tumor evolution. However, it is not clear what the role of PGE2 is in melanoma, and if this complex cytokine interaction is going to occur in the same way. In fact, it has recently been shown that IL-23 plays a dual role in the antitumor immune response in melanoma, depending on tumor immunogenicity (56).

Another important parameter that can influence the type of response generated is the time and kinetics to which the treatments are added. In this work, we chose to use both treatments simultaneously; however, some experimental data show that these factors can have an effect on the type of response generated (57). Moreover, our results showed heterogeneity in the modulation of PD-L1 expression in the tumor microenvironment related with the tumor model. Similarly, Grasselly et al. reported that the antitumor activity of cytotoxic chemotherapy combined with immune checkpoint inhibitors was model-dependent (57).

The protective activity of some polyphenols has been previously reported (39, 58). However, there are few studies where its protective potential in co-treatment with immune checkpoints blockade has been evaluated (38). In previous studies of our group, we have observed the potential of P2Et in the induction of a specific antitumor immune response in both B16-F10 (11, 20) and 4T1 (12, 13, 20) models. We have also reported a preferential migration of CD45^+^ cells to the tumor and peripheral lymphoid organs, allowing a better antigenic presentation through the activation of DC and the decrease of MDSCs and regulatory T cell suppressors (11, 20). These findings suggest that P2Et can regulate PD-L1 expression both directly acting on tumor cells, as previously exposed, but also by the way of immune system modulation. It could not be ruled out that other immune checkpoint inhibitors, similarly regulated, might be modulated by this extract, favoring immune response activation which might be evaluated. In this way, effectiveness of the clinical response to the blockade of PD-L1 and PD-1 has been associated with an increase in TILs (5–7, 12, 23), and in this work we have shown that the use of P2Et favored the intra-tumoral and peripheral activation of the immune response.

Moreover, in both models we observed that treatment with P2Et alone or in combination with anti PD-L1 decreases the frequency of intratumoral MDSC-LC (20). The MDSC are currently presented as one of the main immunosuppressive elements of the antitumor response (59). Indeed, the antitumor activity of the polyphenols through the inhibition of the suppressive function of the MDSC and other suppressor mechanisms has been evidenced for the epigallocatechin gallate and the curcuma, among others (60, 61). Other important effect of P2Et in B16-F10 tumor-bearing mice was the increase of CD8_+_ T cells frequency but the decrease of the frequency of CD8^+^ T cells expressing PD-1, suggesting that PD-1 low/neg cells are expanding upon the treatment.

We demonstrate that mice receiving combined therapy had strengthened T cell response. In tumor tissues, most of the infiltrated T cells displayed an activated phenotype, which may be correlated with a better effector response. In the spleen, cytotoxic T cells showed greater cytotoxic activity and, consequently, may be involved with better control of tumor progression in both models. Comparable results have been reported using a curcumin analogue in combination with anti-PD-L1 in murine bladder cancer (38), perhaps because curcumin has been shown to stimulate the immune response (62), as well as P2Et extract (11, 12, 20).

In summary, these findings suggest that P2Et extract sensitizes B16-F10 cells by increasing PD-L1 expression for an enhanced response to PD-L1 blockade, effect not found in murine breast cancer model, achieving that combination treatment improve antitumor response in the melanoma model by enhancing CD8^+^ T cells activity and suppressing MDSCs.

## Data Availability Statement

The raw data supporting the conclusions of this article will be made available by the authors without undue reservation.

## Ethics Statement

The animal study was reviewed and approved by the ethics committee of the Faculty of Sciences, Pontifical Javeriana University and Animal experimentation committee of Pontifical Javeriana University.

## Author Contributions

PL and AG-C designed and executed the experiments, and acquired and interpreted the data. PL and SF drafted the manuscript. CU developed the *in vivo* animal experiments, acquired and interpreted the data. AB, AD, AM-U, and PR interpreted and analyzed the data. SF leader of the project and designed the experiments, interpreted the results, and revised the manuscript. All authors contributed to the article and approved the submitted version.

## Funding

Funding was provided by the Sistema General de Regalías (BPIN: 2013000100196; contract number 1027-1-2015) and Pontificia Universidad Javeriana. PL was funded by the Departamento Administrativo de Ciencia, Tecnología e Innovación COLCIENCIAS, convocatoria 811-2018 (No. 335-2019; C160I003100000880) and Vicerrectoría de Investigaciones, Pontificia Universidad Javeriana (PUJ-04298-19, contrato de recuperación contingente No. 80740-335-2019), Bogotá, Colombia.

## Conflict of Interest

SF and CU are inventors of a granted patent related to P2Et. SF, PL, AG-C, CU, PR, and AB are inventors of a granted patent related to combined therapy with P2Et and anti-PD-L1 therapy and modulation of PD-L1 expression by P2Et extract.

The remaining authors declare that the research was conducted in the absence of any commercial or financial relationships that could be construed as a potential conflict of interest.

## Abbreviations

TILs, tumor infiltrating lymphocytes; PD-1, programmed cell death protein-1; PD-L1, ligand of PD-1; FDA, Food and Drug Administration; IFN**γ**; Interferon gamma; ATP, adenosine triphosphate; NK, Natural killer; Treg, Regulatory T cells; MDSCs, myeloid-derived suppressor cells; GAPDH, Gliceraldehído-3-fosfato deshidrogenasa; HIF-1*α*, Hypoxia-inducible factor-1α; DCs, Dendritic cells; cDCs, Conventional Dendritic cells; TDLNs, tumor-draining lymph nodes; MDSC-LCs, Myeloid-derived suppressor like cells; M-MDSC, monocytic MDSC; PMN-MDSC, Polymorphonuclear MDSC; CoCl_2_, Cobaltous chloride.
