# Supplementary material for: An Immunomodulatory Gallotanin-Rich Fraction From Caesalpinia spinosa Enhances the Therapeutic Effect of Anti-PD-L1 in Melanoma
Source: Front Immunol. 2020 Nov 18;11:584959. doi: 10.3389/fimmu.2020.584959 (PMC7708328; doi:10.3389/fimmu.2020.584959)
Supplement: Supplementary file 1 [file Presentation_1.pdf]

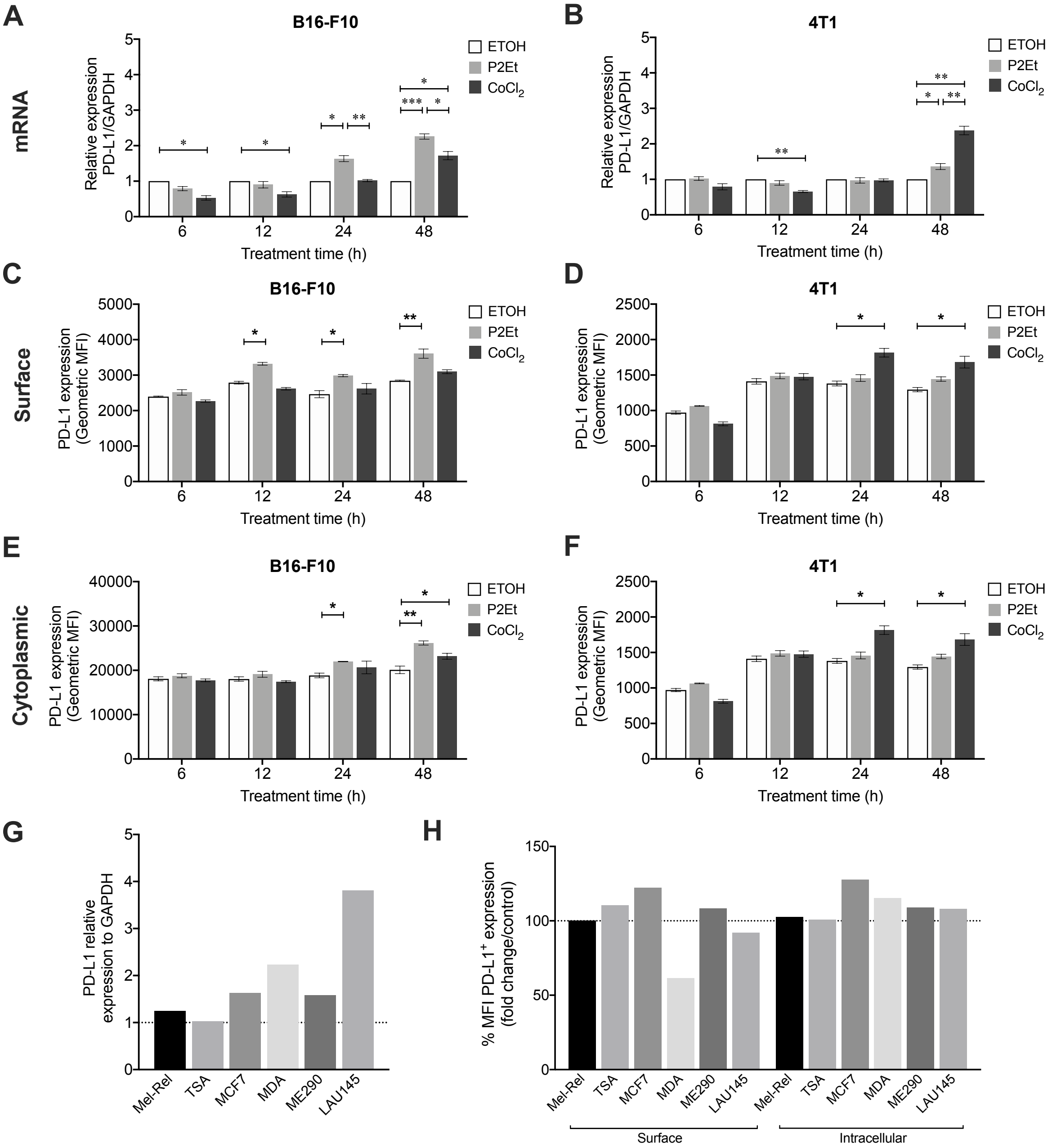

Supplementary Figure 1. Effect of P2Et treatment on PD-L1 expression in cell lines. PD-L1 relative expression to GAPDH gene in B16-F10 **(A)** and 4T1 **(B)** cultured cells during 6, 12, 24 and 48 hours with one-half of the P2Et IC_{50} extract, cobaltous chloride ($CoCl_2$) or ethanol (EtOH) analyzed by qRT-PCR and quantitated as a fold change against the control by using the $2^{-\Delta\Delta CT}$ method. Geometric Mean Fluorescence Intensity (MFI) of surface **(C and D)** or intracellular **(E and F)** PD-L1 expression in B16-F10 **(C and E)** and 4T1 **(D and F)** cells. **(G)** PD-L1 relative expression to GAPDH gene in cells treated during 48 hours with one-half of the P2Et IC_{50} extract analyzed by qRT-PCR and quantitated as a fold change against the control (EtOH) by using the $2^{-\Delta\Delta CT}$ method. **(H)** MFI of surface or intracellular PD-L1 expression represented as fold change against to the control (EtOH). In all cases data

are represented as the mean ± SEM. The p values were calculated using a Mann-Whitney U test or Kruskal-Wallis test with Dunn's post-test. $^{*}p < 0.05$, $^{**}p < 0.01$, $^{***}p < 0.001$.

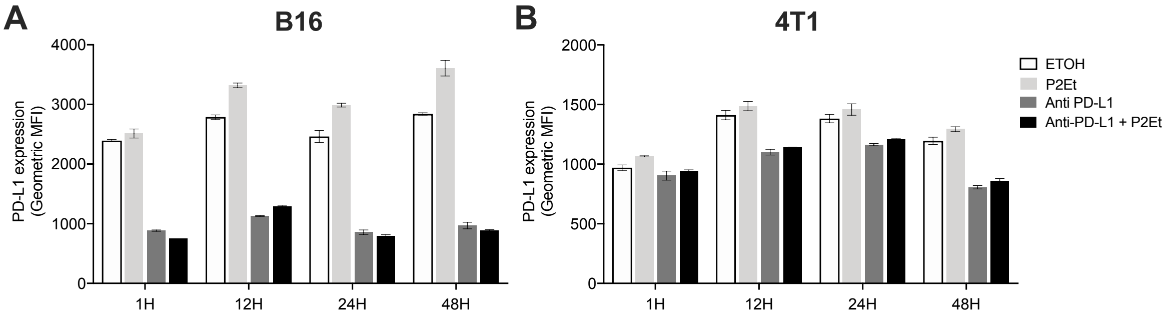

Supplementary Figure 2. In vitro staining of surface PD-L1 after adding 10X more the therapeutic anti-PD-L1 antibody. Geometric Mean Fluorescence Intensity (MFI) of surface PD-L1 expression in B16-F10 **(A)** and 4T1 **(B)** cells at 1, 12, 24 or 48 hours after in vitro treatment with P2Et, therapeutic anti-PD-L1 or anti-PD-L1 + P2Et.

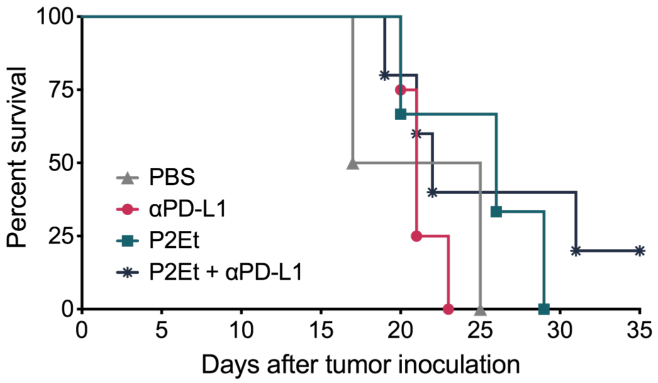

Supplementary Figure 3. Survival analysis in B16-F10 tumor-bearing mice treated with αPD-L1, P2Et, P2Et plus αPD-L1 or PBS as control.

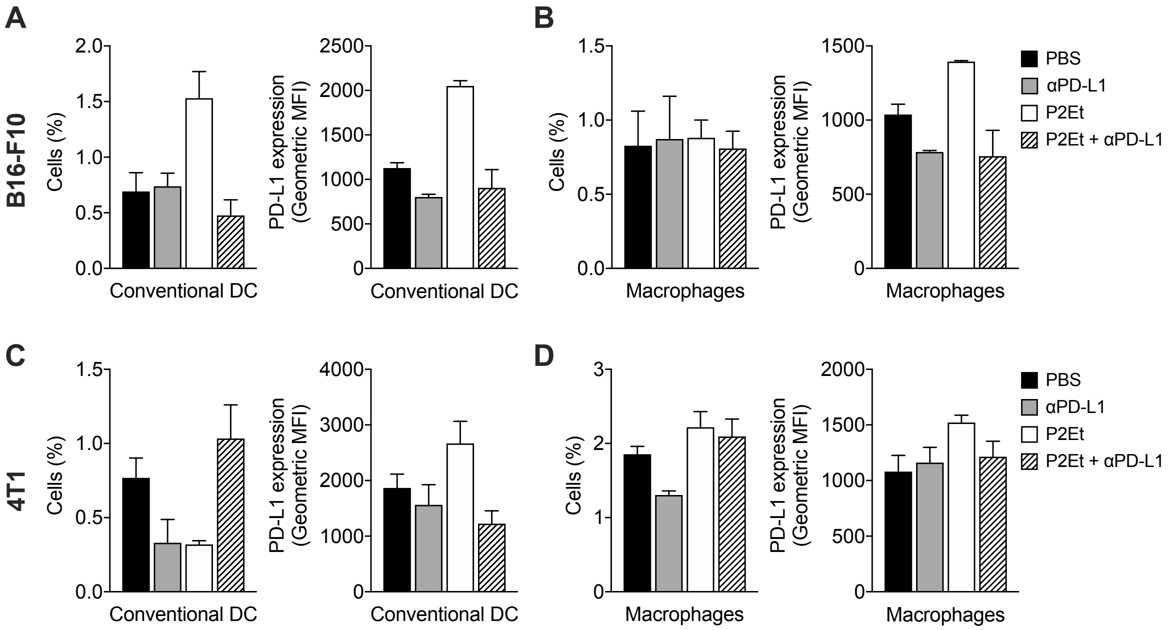

Supplementary Figure 4. Effect of treatments in conventional dendritic cells and macrophages from tumor-draining lymph nodes. (A) Frequency of conventional DC and Geometric Mean Fluorescence Intensity (MFI) of PD-L1 expression in cDC from B16 tumor bearing-mice. **(B)** Frequency of macrophages and geometric MFI of PD-L1 expression in macrophages from B16 tumor bearing-mice (n=3). **(C)** Frequency of conventional DC and Geometric Mean Fluorescence Intensity (MFI) of PD-L1 expression in cDC from 4T1 tumor bearing-mice. **(D)** Frequency of macrophages and geometric MFI of PD-L1 expression in macrophages from 4T1 tumor bearing-mice (n=3). In all cases data are represented as the mean ± SEM.

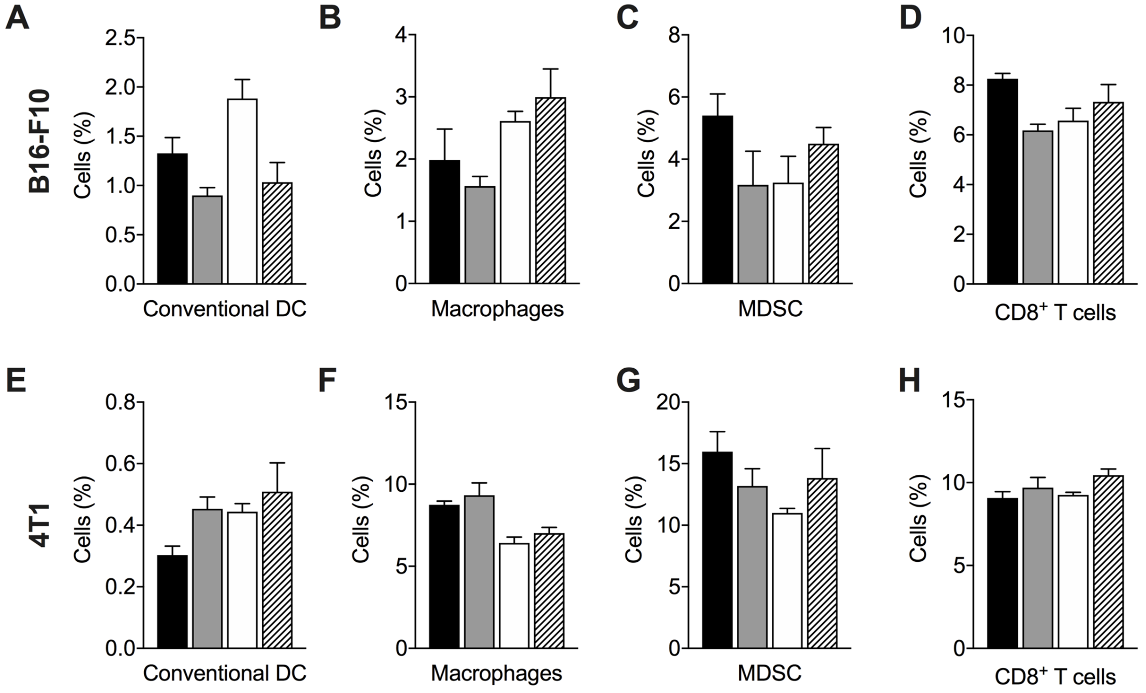

Supplementary Figure 5. Effect of treatments in conventional dendritic cells, macrophages, MDSC and $CD8^+$ T cells from spleen. Frequency of conventional DC **(A)**, macrophages **(B),** MDSC **(C)**, and $CD8^+$ T cells **(D)** from B16 tumor bearing-mice (n=3) treated with PBS, P2Et, therapeutic anti-PD-L1 or anti-PD-L1 + P2Et. Frequency conventional DC **(E)**, macrophages **(F),** MDSC **(G)**, and $CD8^+$ T cells **(H)** from 4T1 tumor bearing-mice (n=3) treated with PBS, P2Et, therapeutic anti-PD-L1 or anti-PD-L1 + P2Et. In all cases data are represented as the mean ± SEM.

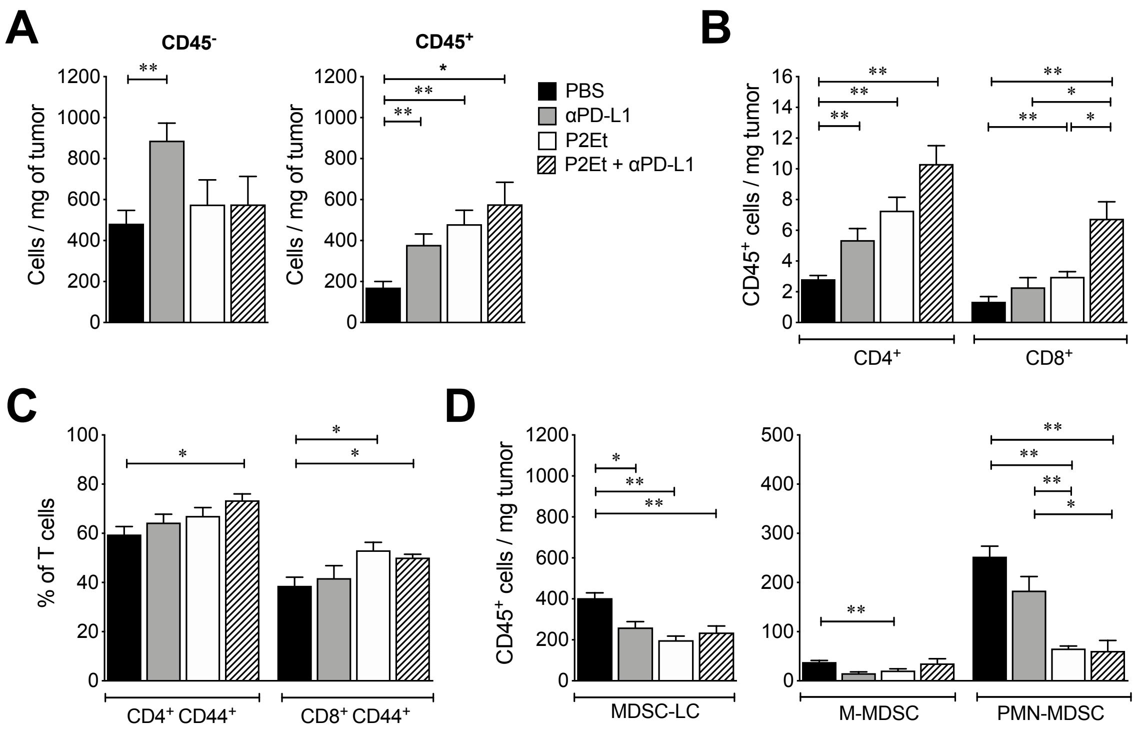

Supplementary Figure 6. P2Et, αPD-L1 or P2Et plus αPD-L1 treatments modulates the immune response in breast 4T1 model. **(A)** Absolute numbers of $CD45^-$ and $CD45^+$ cells per mg of tumor in mice treated with αPD-L1, P2Et, P2Et plus αPD-L1 or PBS (control). **(B)** Absolute numbers of $CD4^+$ and $CD8^+$ T cells per mg of tumor in each group of treated mice. **(C)** Frequency of activated $CD44^+$ $CD4^+$ and $CD8^+$ T cells in tumor from each group of treated mice. **(D)** Number of MDSC-LC, M-MDSC and PMN-MDSC per mg of tumor from αPD-L1, P2Et, P2Et plus αPD-L1 or PBS treated mice. In all cases data are represented as the mean ± SEM. $*p < 0.05$, $**p < 0.01$.
